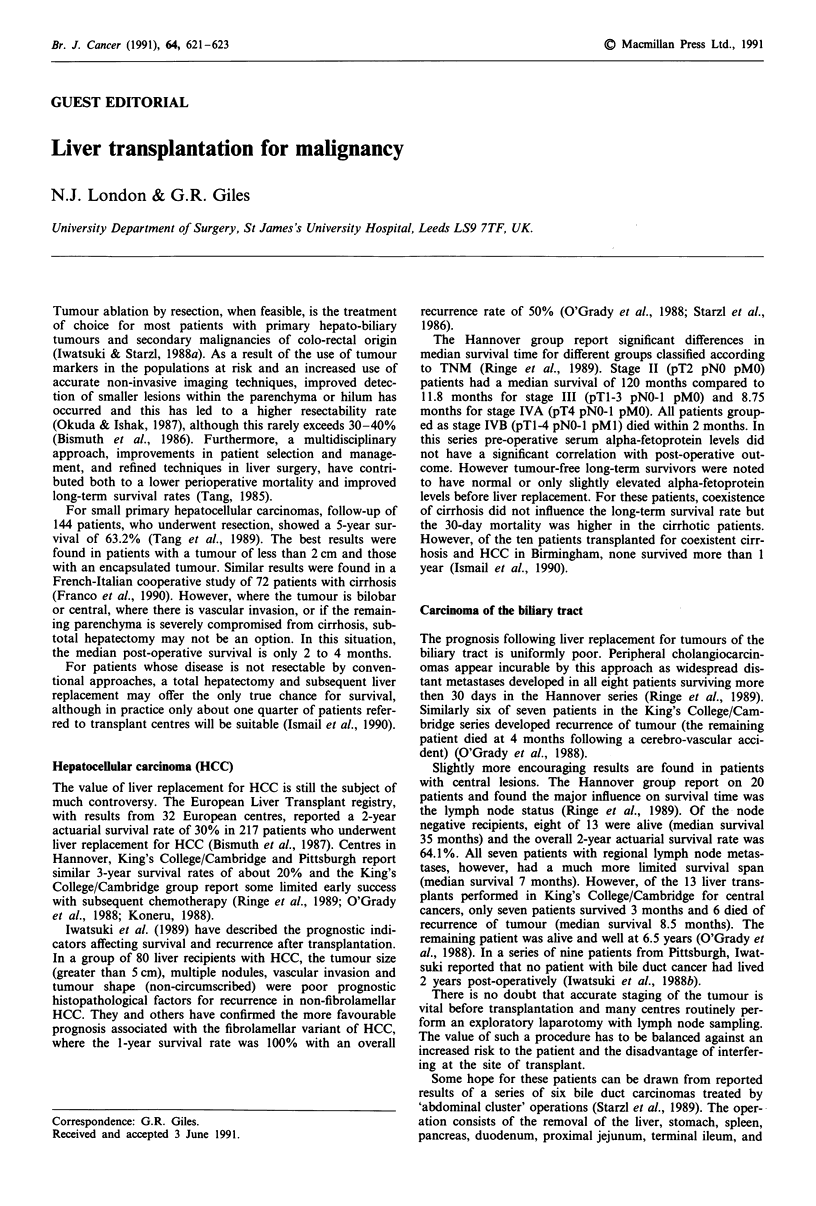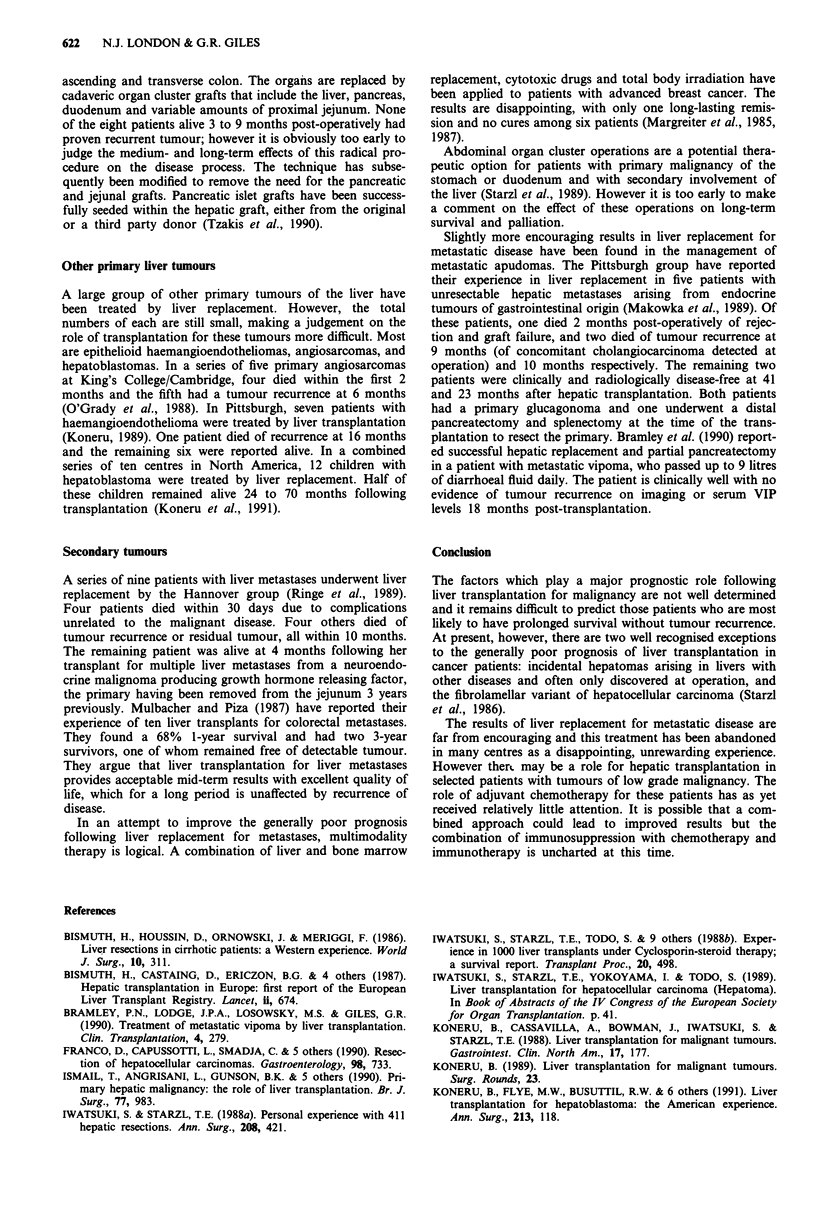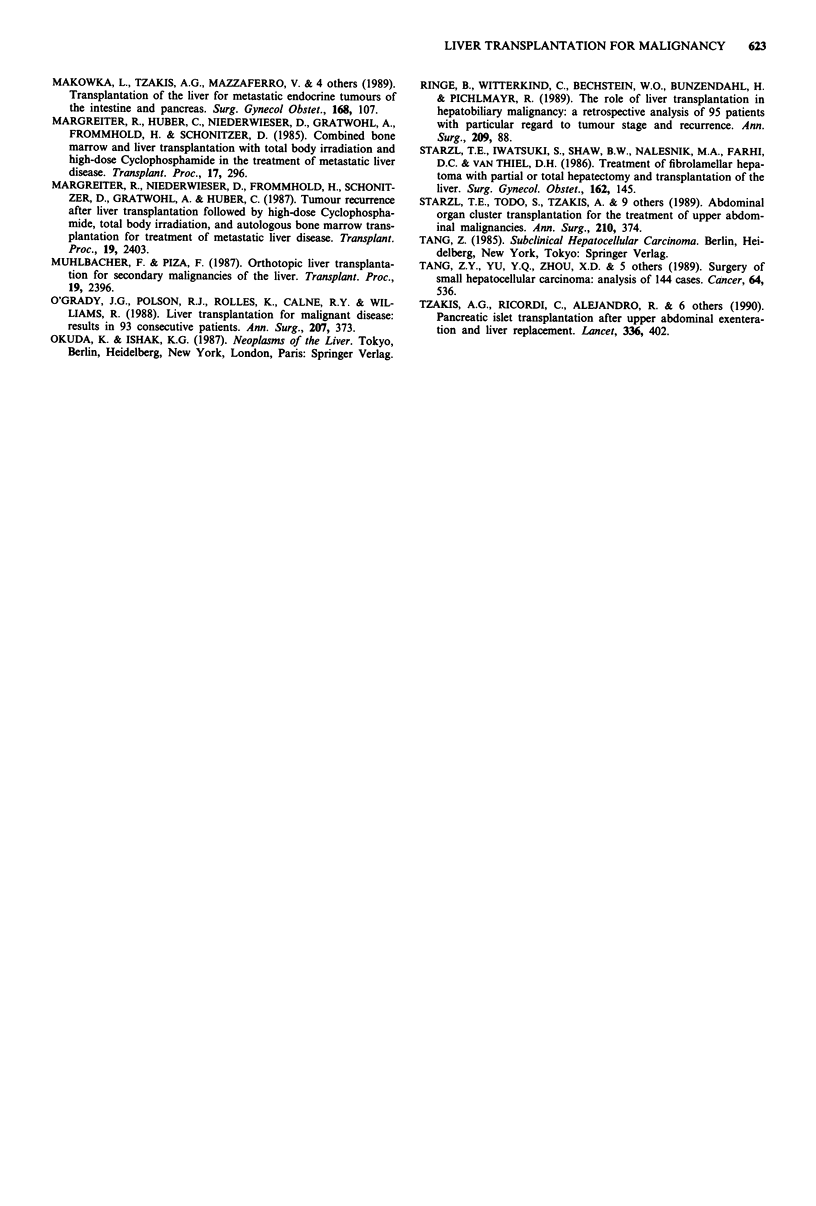# Liver transplantation for malignancy.

**DOI:** 10.1038/bjc.1991.373

**Published:** 1991-10

**Authors:** N. J. London, G. R. Giles


Br. J. Cancer (1991), 64, 621–623 © Macmillan Press Ltd., 1991

GUEST EDITORIAL

### Liver transplantation for malignancy

N.J. London & G.R. Giles

*University Department of Surgery, St James's University Hospital, Leeds LS9 7TF, UK.*

Tumour ablation by resection, when feasible, is the treatment of choice for most patients with primary hepato-biliary tumours and secondary malignancies of colo-rectal origin (Iwatsuki & Starzl, 1988*a*). As a result of the use of tumour markers in the populations at risk and an increased use of accurate non-invasive imaging techniques, improved detection of smaller lesions within the parenchyma or hilum has occurred and this has led to a higher resectability rate (Okuda & Ishak, 1987), although this rarely exceeds 30–40% (Bismuth *et al.*, 1986). Furthermore, a multidisciplinary approach, improvements in patient selection and management, and refined techniques in liver surgery, have contributed both to a lower perioperative mortality and improved long-term survival rates (Tang, 1985).

For small primary hepatocellular carcinomas, follow-up of 144 patients, who underwent resection, showed a 5-year survival of 63.2% (Tang *et al.*, 1989). The best results were found in patients with a tumour of less than 2 cm and those with an encapsulated tumour. Similar results were found in a French-Italian cooperative study of 72 patients with cirrhosis (Franco *et al.*, 1990). However, where the tumour is bilobar or central, where there is vascular invasion, or if the remaining parenchyma is severely compromised from cirrhosis, subtotal hepatectomy may not be an option. In this situation, the median post-operative survival is only 2 to 4 months.

For patients whose disease is not resectable by conventional approaches, a total hepatectomy and subsequent liver replacement may offer the only true chance for survival, although in practice only about one quarter of patients referred to transplant centres will be suitable (Ismail *et al.*, 1990).

### Hepatocellular carcinoma (HCC)

The value of liver replacement for HCC is still the subject of much controversy. The European Liver Transplant registry, with results from 32 European centres, reported a 2-year actuarial survival rate of 30% in 217 patients who underwent liver replacement for HCC (Bismuth *et al.*, 1987). Centres in Hannover, King's College/Cambridge and Pittsburgh report similar 3-year survival rates of about 20% and the King's College/Cambridge group report some limited early success with subsequent chemotherapy (Ringe *et al.*, 1989; O'Grady *et al.*, 1988; Koneru, 1988).

Iwatsuki *et al.* (1989) have described the prognostic indicators affecting survival and recurrence after transplantation. In a group of 80 liver recipients with HCC, the tumour size (greater than 5 cm), multiple nodules, vascular invasion and tumour shape (non-circumscribed) were poor prognostic histopathological factors for recurrence in non-fibrolamellar HCC. They and others have confirmed the more favourable prognosis associated with the fibrolamellar variant of HCC, where the 1-year survival rate was 100% with an overall recurrence rate of 50% (O'Grady *et al.*, 1988; Starzl *et al.*, 1986).

The Hannover group report significant differences in median survival time for different groups classified according to TNM (Ringe *et al.*, 1989). Stage II (pT2 pN0 pM0) patients had a median survival of 120 months compared to 11.8 months for stage III (pT1-3 pN0-1 pM0) and 8.75 months for stage IVA (pT4 pN0-1 pM0). All patients grouped as stage IVB (pT1-4 pN0-1 pM1) died within 2 months. In this series pre-operative serum alpha-fetoprotein levels did not have a significant correlation with post-operative outcome. However tumour-free long-term survivors were noted to have normal or only slightly elevated alpha-fetoprotein levels before liver replacement. For these patients, coexistence of cirrhosis did not influence the long-term survival rate but the 30-day mortality was higher in the cirrhotic patients. However, of the ten patients transplanted for coexistent cirrhosis and HCC in Birmingham, none survived more than 1 year (Ismail *et al.*, 1990).

### Carcinoma of the biliary tract

The prognosis following liver replacement for tumours of the biliary tract is uniformly poor. Peripheral cholangiocarcinomas appear incurable by this approach as widespread distant metastases developed in all eight patients surviving more then 30 days in the Hannover series (Ringe *et al.*, 1989). Similarly six of seven patients in the King's College/Cambridge series developed recurrence of tumour (the remaining patient died at 4 months following a cerebro-vascular accident) (O'Grady *et al.*, 1988).

Slightly more encouraging results are found in patients with central lesions. The Hannover group report on 20 patients and found the major influence on survival time was the lymph node status (Ringe *et al.*, 1989). Of the node negative recipients, eight of 13 were alive (median survival 35 months) and the overall 2-year actuarial survival rate was 64.1%. All seven patients with regional lymph node metastases, however, had a much more limited survival span (median survival 7 months). However, of the 13 liver transplants performed in King's College/Cambridge for central cancers, only seven patients survived 3 months and 6 died of recurrence of tumour (median survival 8.5 months). The remaining patient was alive and well at 6.5 years (O'Grady *et al.*, 1988). In a series of nine patients from Pittsburgh, Iwatsuki reported that no patient with bile duct cancer had lived 2 years post-operatively (Iwatsuki *et al.*, 1988*b*).

There is no doubt that accurate staging of the tumour is vital before transplantation and many centres routinely perform an exploratory laparotomy with lymph node sampling. The value of such a procedure has to be balanced against an increased risk to the patient and the disadvantage of interfering at the site of transplant.

Some hope for these patients can be drawn from reported results of a series of six bile duct carcinomas treated by 'abdominal cluster' operations (Starzl *et al.*, 1989). The operation consists of the removal of the liver, stomach, spleen, pancreas, duodenum, proximal jejunum, terminal ileum, and

Correspondence: G.R. Giles.
Received and accepted 3 June 1991.

ascending and transverse colon. The organs are replaced by cadaveric organ cluster grafts that include the liver, pancreas, duodenum and variable amounts of proximal jejunum. None of the eight patients alive 3 to 9 months post-operatively had proven recurrent tumour; however it is obviously too early to judge the medium- and long-term effects of this radical procedure on the disease process. The technique has subsequently been modified to remove the need for the pancreatic and jejunal grafts. Pancreatic islet grafts have been successfully seeded within the hepatic graft, either from the original or a third party donor (Tzakis *et al.*, 1990).

### Other primary liver tumours

A large group of other primary tumours of the liver have been treated by liver replacement. However, the total numbers of each are still small, making a judgement on the role of transplantation for these tumours more difficult. Most are epithelioid haemangioendotheliomas, angiosarcomas, and hepatoblastomas. In a series of five primary angiosarcomas at King's College/Cambridge, four died within the first 2 months and the fifth had a tumour recurrence at 6 months (O'Grady *et al.*, 1988). In Pittsburgh, seven patients with haemangioendothelioma were treated by liver transplantation (Koneru, 1989). One patient died of recurrence at 16 months and the remaining six were reported alive. In a combined series of ten centres in North America, 12 children with hepatoblastoma were treated by liver replacement. Half of these children remained alive 24 to 70 months following transplantation (Koneru *et al.*, 1991).

### Secondary tumours

A series of nine patients with liver metastases underwent liver replacement by the Hannover group (Ringe *et al.*, 1989). Four patients died within 30 days due to complications unrelated to the malignant disease. Four others died of tumour recurrence or residual tumour, all within 10 months. The remaining patient was alive at 4 months following her transplant for multiple liver metastases from a neuroendocrine malignoma producing growth hormone releasing factor, the primary having been removed from the jejunum 3 years previously. Mulbacher and Piza (1987) have reported their experience of ten liver transplants for colorectal metastases. They found a 68% 1-year survival and had two 3-year survivors, one of whom remained free of detectable tumour. They argue that liver transplantation for liver metastases provides acceptable mid-term results with excellent quality of life, which for a long period is unaffected by recurrence of disease.

In an attempt to improve the generally poor prognosis following liver replacement for metastases, multimodality therapy is logical. A combination of liver and bone marrow replacement, cytotoxic drugs and total body irradiation have been applied to patients with advanced breast cancer. The results are disappointing, with only one long-lasting remission and no cures among six patients (Margreiter *et al.*, 1985, 1987).

Abdominal organ cluster operations are a potential therapeutic option for patients with primary malignancy of the stomach or duodenum and with secondary involvement of the liver (Starzl *et al.*, 1989). However it is too early to make a comment on the effect of these operations on long-term survival and palliation.

Slightly more encouraging results in liver replacement for metastatic disease have been found in the management of metastatic apudomas. The Pittsburgh group have reported their experience in liver replacement in five patients with unresectable hepatic metastases arising from endocrine tumours of gastrointestinal origin (Makowka *et al.*, 1989). Of these patients, one died 2 months post-operatively of rejection and graft failure, and two died of tumour recurrence at 9 months (of concomitant cholangiocarcinoma detected at operation) and 10 months respectively. The remaining two patients were clinically and radiologically disease-free at 41 and 23 months after hepatic transplantation. Both patients had a primary glucagonoma and one underwent a distal pancreatectomy and splenectomy at the time of the transplantation to resect the primary. Bramley *et al.* (1990) reported successful hepatic replacement and partial pancreatectomy in a patient with metastatic vipoma, who passed up to 9 litres of diarrhoeal fluid daily. The patient is clinically well with no evidence of tumour recurrence on imaging or serum VIP levels 18 months post-transplantation.

### Conclusion

The factors which play a major prognostic role following liver transplantation for malignancy are not well determined and it remains difficult to predict those patients who are most likely to have prolonged survival without tumour recurrence. At present, however, there are two well recognised exceptions to the generally poor prognosis of liver transplantation in cancer patients: incidental hepatomas arising in livers with other diseases and often only discovered at operation, and the fibrolamellar variant of hepatocellular carcinoma (Starzl *et al.*, 1986).

The results of liver replacement for metastatic disease are far from encouraging and this treatment has been abandoned in many centres as a disappointing, unrewarding experience. However there may be a role for hepatic transplantation in selected patients with tumours of low grade malignancy. The role of adjuvant chemotherapy for these patients has as yet received relatively little attention. It is possible that a combined approach could lead to improved results but the combination of immunosuppression with chemotherapy and immunotherapy is uncharted at this time.

### References

BISMUTH, H., HOUSSIN, D., ORNOWSKI, J. & MERIGGI, F. (1986). Liver resections in cirrhotic patients: a Western experience. *World J. Surg.*, **10**, 311.

BISMUTH, H., CASTAING, D., ERICZON, B.G. & 4 others (1987). Hepatic transplantation in Europe: first report of the European Liver Transplant Registry. *Lancet*, **ii**, 674.

BRAMLEY, P.N., LODGE, J.P.A., LOSOWSKY, M.S. & GILES, G.R. (1990). Treatment of metastatic vipoma by liver transplantation. *Clin. Transplantation*, **4**, 279.

FRANCO, D., CAPUSSOTTI, L., SMADJA, C. & 5 others (1990). Resection of hepatocellular carcinomas. *Gastroenterology*, **98**, 733.

ISMAIL, T., ANGRISANI, L., GUNSON, B.K. & 5 others (1990). Primary hepatic malignancy: the role of liver transplantation. *Br. J. Surg.*, **77**, 983.

IWATSUKI, S. & STARZL, T.E. (1988*a*). Personal experience with 411 hepatic resections. *Ann. Surg.*, **208**, 421.

IWATSUKI, S., STARZL, T.E., TODO, S. & 9 others (1988*b*). Experience in 1000 liver transplants under Cyclosporin-steroid therapy; a survival report. *Transplant Proc.*, **20**, 498.

IWATSUKI, S., STARZL, T.E., YOKOYAMA, I. & TODO, S. (1989). Liver transplantation for hepatocellular carcinoma (Hepatoma). In *Book of Abstracts of the IV Congress of the European Society for Organ Transplantation*. p. 41.

KONERU, B., CASSAVILLA, A., BOWMAN, J., IWATSUKI, S. & STARZL, T.E. (1988). Liver transplantation for malignant tumours. *Gastrointest. Clin. North Am.*, **17**, 177.

KONERU, B. (1989). Liver transplantation for malignant tumours. *Surg. Rounds*, **23**.

KONERU, B., FLYE, M.W., BUSUTTIL, R.W. & 6 others (1991). Liver transplantation for hepatoblastoma: the American experience. *Ann. Surg.*, **213**, 118.

MAKOWKA, L., TZAKIS, A.G., MAZZAFERRO, V. & 4 others (1989). Transplantation of the liver for metastatic endocrine tumours of the intestine and pancreas. *Surg. Gynecol Obstet.*, **168**, 107.

MARGREITER, R., HUBER, C., NIEDERWIESER, D., GRATWOHL, A., FROMMHOLD, H. & SCHONITZER, D. (1985). Combined bone marrow and liver transplantation with total body irradiation and high-dose Cyclophosphamide in the treatment of metastatic liver disease. *Transplant. Proc.*, **17**, 296.

MARGREITER, R., NIEDERWIESER, D., FROMMHOLD, H., SCHONITZER, D., GRATWOHL, A. & HUBER, C. (1987). Tumour recurrence after liver transplantation followed by high-dose Cyclophosphamide, total body irradiation, and autologous bone marrow transplantation for treatment of metastatic liver disease. *Transplant. Proc.*, **19**, 2403.

MUHLBACHER, F. & PIZA, F. (1987). Orthotopic liver transplantation for secondary malignancies of the liver. *Transplant. Proc.*, **19**, 2396.

O'GRADY, J.G., POLSON, R.J., ROLLES, K., CALNE, R.Y. & WILLIAMS, R. (1988). Liver transplantation for malignant disease: results in 93 consecutive patients. *Ann. Surg.*, **207**, 373.

OKUDA, K. & ISHAK, K.G. (1987). *Neoplasms of the Liver*. Tokyo, Berlin, Heidelberg, New York, London, Paris: Springer Verlag.

RINGE, B., WITTERKIND, C., BECHSTEIN, W.O., BUNZENDAHL, H. & PICHLMAYR, R. (1989). The role of liver transplantation in hepatobiliary malignancy: a retrospective analysis of 95 patients with particular regard to tumour stage and recurrence. *Ann. Surg.*, **209**, 88.

STARZL, T.E., IWATSUKI, S., SHAW, B.W., NALESNIK, M.A., FARHI, D.C. & VAN THIEL, D.H. (1986). Treatment of fibrolamellar hepatoma with partial or total hepatectomy and transplantation of the liver. *Surg. Gynecol. Obstet.*, **162**, 145.

STARZL, T.E., TODO, S., TZAKIS, A. & 9 others (1989). Abdominal organ cluster transplantation for the treatment of upper abdominal malignancies. *Ann. Surg.*, **210**, 374.

TANG, Z. (1985). *Subclinical Hepatocellular Carcinoma*. Berlin, Heidelberg, New York, Tokyo: Springer Verlag.

TANG, Z.Y., YU, Y.Q., ZHOU, X.D. & 5 others (1989). Surgery of small hepatocellular carcinoma: analysis of 144 cases. *Cancer*, **64**, 536.

TZAKIS, A.G., RICORDI, C., ALEJANDRO, R. & 6 others (1990). Pancreatic islet transplantation after upper abdominal exenteration and liver replacement. *Lancet*, **336**, 402.